# Scientific Symposium “Small Solution for Big Water-Related Problems: Innovative Microarrays and Small Sensors to Cope with Water Quality and Food Security”

**DOI:** 10.3390/ijerph121214992

**Published:** 2015-12-04

**Authors:** Stefania Marcheggiani, Roberto Spurio, Lucia Cimarelli, Duarte Tito, Laura Mancini

**Affiliations:** 1 Environmental, Quality and Fishfarm Unit, Environment & Primary Prevention Department, Istituto Superiore di Sanità, Viale Regina Elena 299, 00161 Rome, Italy; laura.mancini@iss.it; 2 Laboratory of Genetics, School of Biosciences and Veterinary Medicine, University of Camerino, 62032 Camerino, Italy; roberto.spurio@unicam.it (R.S.); lucia.cimarelli@unicam.it (L.C.); 3 Elysium Projects Ltd., Stanton, Glyn Garth, LL59 5PE Anglesey, Wales, UK; duarte.tito@elysiumprojects.eu

**Keywords:** water quality, emerging pathogens, public health, endocrine disrupting compounds, microchip technology

## Abstract

This issue presents the conclusive results of two European Commission funded Projects, namely Universal Microarrays for the Evaluation of Fresh-water Quality Based on Detection of Pathogens and their Toxins (MicroAQUA) and Rationally Designed Aquatic Receptors (RADAR). These projects focused their activities on the quality of drinking water as an extremely important factor for public health of humans and animals. The MicroAQUA Project aimed at developing a universal microarray chip for the detection of various pathogens (cyanobacteria, bacteria, viruses and parasitic protozoa) and their toxins in waters. In addition, the project included the detection of select species of diatoms, which represent reliable bio-indicators to assess overall water quality. Large numbers of compounds are released into the environment; some of these are toxins such as endocrine disrupting compounds (EDCs) and can affect the endocrine, immune and nervous systems of a wide range of animals causing alterations such as reproductive disorders and cancer. Detection of these contaminants in water systems is important to protect sensitive environmental sites and reduce the risk of toxins entering the food chain. A modular platform for monitoring toxins in water and food production facilities, using biosensors derived from aquatic organisms, was the main goal of RADAR Project.

## 1. Introduction

An International symposium co-organized by the two EC consortia, with the aim to disseminate the results obtained by the µAQUA and RADAR research teams, took place in Rome, in October 2014, at the Department of the Environment and Primary Prevention of the Italian National Institute for Health (Istituto Superiore di Sanità, ISS). The meeting agenda included a cluster of different issues such as the microchip technology applied to water quality evaluation; rapid and efficient methods to monitor the presence of emerging and re-emerging pathogens in aquatic ecosystems; aquatic biosensors; toxins detection in freshwater; detection of endocrine disrupting chemicals in water and drinks such as milk and fruit juices; cyanophages and the influence of climate change on ecosystems.

There is a close relationship between the quality of fresh and marine waters and human health. This relationship stems primarily from the direct or indirect consumption of water polluted by toxic chemicals and/or contaminated by pathogenic organisms. Monitoring water quality is therefore of paramount importance for safeguarding public health. Efficient primary preventive measures are required to cope with the rapid spread of pathologies linked to the progressive deterioration of the environment. 

It is commonly recognized that biological indicators can provide useful information for water quality assessment. The community structure (abundance and composition) of macroinvertebrates, diatoms, macrophytes and fish must be analyzed and results expressed as ecological quality status (EQS) using official metrics or indices, which are then compared to reference conditions [1].

In this context, Tancioni *et al*., [2] proposed a study where the use of gonadal alterations in the thinlip grey mullet (*Liza ramada*) was evaluated as a biological indicator. The reproductive status associated to the presence of gonadal alterations were studied in 206 mullets. Intersex and irregularly shaped gonads of this species were observed in the most polluted sites. The results of this study suggest that *L. ramada* may be used as a sentinel species in environmental risk assessment and support the use of gonadal alterations as bioindicator for extensive monitoring of pollution in lower stretches of rivers and estuarine areas. 

A study reported in this issue addresses the effect of diuron, a herbicide frequently found in surface and groundwater, on *Saccharomyces cerevisiae* used as biosensor model for toxicological evaluation [3]. The authors found that the yeast-based probes had enough sensitivity to detect the pesticide in drinking water.

## 2. Microchip Technology Applied to Water Quality Evaluation

Microarray technology allows the simultaneous analysis of thousands of genes after hybridization of labelled DNA or total RNA with a substrate containing thousands of oligo- nucleotides [4]. The fluorescence detected at each spot of the microchip represents the signal derived from the base-pairing between complementary nucleic acid sequences (target and probe). Some possible shortcoming of this technique are the difficulties to distinguish between viable and not-viable cells, the relatively high costs, the presence of unspecific hybridization signals and the need of a validation method [5].

The molecular methods developed by the MicroAQUA consortium, mainly through the rational use of specific molecular probes combined in a universal microarray chip, are described in three articles [6,7,8]. The paper by Dhar *et al.* investigates a specific diatom of the *Amphora* genus, namely *Amphora coffeaeformis*, isolated from the estuarine waters of the Tyrrhenian coast of Central Italy [6]. 

The link between this diatom species and the severe intoxication after the consumption of cultured shellfish was already described in 1987. The presence in the Canadian marine waters of *A. coffeaeformis*, as well as of cells belonging to the *Pseudo-nitzschia* diatom genus, was associated with the production of the neurotoxic compound domoic acid from both diatom species and the intoxication episode [9,10,11].

To cope with the need to detect rapidly the presence of *A. coffeaeformis* cells in estuarine environment water samples, the authors developed a molecular tool based on species-specific oligonucleotide probe design and selection of probes capable of recognizing with good sensitivity the presence of this diatom when tested in microarray hybridization experiments.

Because the nature of diatom communities reflects environmental conditions and since they react sensitively to water quality changes, these microorganisms have become widely used over the last 50 years as biomarkers for water quality assessment. In this context, the paper by Cimarelli *et al.* takes into consideration a panel of selected diatom species as bioindicators of different water quality levels [7]. The authors described the procedure, based on the same approach of detection of the potentially toxic *A. coffeaeformis*, to obtain molecular probes able to recognize, by hybridization, nine diatom species commonly found in rivers of the Italian Central-East Apennine area, and selected from the five classes of water quality identified by the European Water Framework Directive.

Because the degree of sequence divergence of the ribosomal RNA does not allow a reliable identification at the species level, the authors followed an alternative approach, consisting in the search and identification of species-specific sequences of protein-encoding genes eEF-1a and SIT. eEF-1a gene encodes for elongation factor, while SIT gene encodes for the diatom-specific silicic acid transporter. These genes represent unexplored targets and, being typically eukaryotic, can be amplified by polymerase chain reaction (PCR) to produce fluorescently labeled DNA targets for microarray hybridization experiments without the risk of contamination with nucleic acids from bacteria inevitably present in the diatom cultures.

## 3. Rapid and Efficient Methods to Monitor the Presence of Emerging and Re-Emerging Pathogens in Aquatic Ecosystems

The World Health Organization (WHO) recognizes that access to adequate water supplies is a fundamental human right [12]. In the last decade, infectious diseases caused by recently identified or previousl known microorganisms have been showing upward trends in incidence or prevalence worldwide [13]. Waterborne and water-related diseases are disease-causing bacteria, viruses, protozoa and helminthes. These potentially emerging or re-emerging pathogens that are transmitted to people when they inhale, contact or ingest untreated or inadequately treated water, are among the most serious threats to public health [14,15]. Rapid identification of pathogens present in water is needed to ensure water quality and human beings safety. Microbiological indicators such as *Escherichia coli* and Enterococci, are widely used in the monitoring programs for regulatory control and in human health risk assessment together with a number of selected pathogens (*i.e.*, *Salmonella* spp., Enteroviruses and *Cryptosporidum*) [16,17].

The current monitoring methods rely on culturing techniques that either measure the growth or the metabolic endpoint of a microorganism after an incubation period. Further, to pinpoint the features of the pathogenic microorganisms selective identification steps are required, which are mostly time-consuming and unsuited when applied to emerging harmful microorganisms [4]. Moreover, the low concentration of pathogens in aquatic ecosystems requires preliminary steps of collection of large volumes of water and enrichment and concentration of the sample for their detection. There is no unified method to encompass the collection and analysis of a water sample for all pathogenic microorganisms of interest [18]. The most important requirements for reliable analysis include: specificity, sensitivity, reproducibility of results, speed, automation and low cost [19]. Many microorganisms are not cultured or can enter in a viable but not culturable (VBNC) state. Therefore, methods focused on immunological or genetic characteristics, which are able to achieve a high degree of sensitivity and specificity play a fundamental role in their detection [20]. In fact, some of these methods permit the detection of specific colourable and/or not colourable microorganisms within hours, instead of the days required by the traditional methods [21]. A summary of methods currently used for waterborne detection is shown in Table 1.

**Table 1:** Methods currently used for the detection of water pathogens.

Counting, Colourimetric and Fluorimetric Methods	Genetic Methods	Immunological Methods
Heterotrophic plate count (HPC test) or Standard Plate Count; Enzyme/substrate methods based on colorimetric or fluorimetric assays.	Polimerase chain reaction (PCR); Multiplex PCR (mPCR); Quantitative PCR (qPCR); Droplet Digital PCR (dd PCR); DNA sequencing; Next generation sequencing (NGS); Microarrays; Biosensors (Antibody microarray); Fluorescence *in situ* hybridization (FISH).	Enzyme-Linked Immuno Sorbent Assay (ELISA); Lateral flow tests (Immuno-chromatographic assays); Surface Plasmon Resonance (SPR); Western blots; Flow cytometry (FCM) paired with immuno-magnetic capture to concentrate cells.

In the field of water monitoring, some commercial kits for the detection of biological contaminants in water samples are available. Most of these assays, performed by the water industry, are based on conventional approaches (enzymatic activity, culturing, *etc*.) but are not highly specific and are usually time consuming. In this context, the paper by Tryland *et al.,* [22] describes the fully automated Colifast ALARM™ for daily monitoring of the presence/absence of *E. coli* in raw water at Oset drinking water treatment plant in Oslo, Norway. *E. coli* was detected in 18% of the daily samples. The results of monitoring supported the hypothesis that warmer winters with shorter periods with ice cover on lakes, which may be a consequence of climate changes, may reduce the hygienic barrier efficiency in deep lakes used as drinking water sources.

### 3.1. Genetic Methods

Novel techniques and molecular tools are now being explored for evaluating and characterizing waterborne pathogens. The polymerase chain reaction (PCR) is one of the most commonly used molecular methods for the detection of many pathogenic microorganisms [23]. Several variations of PCR such as multiplex PCR (mPCR) [23,24] and quantitative real-time PCR (qPCR) have been refined and specifically tailored for this goal [25]. In particular, qPCR provides high sensitivity and specificity, faster rate of detection, minimizes the risk of cross-contamination, and there is no need for post-PCR analysis [26]. The qPCR approach has been used to quantify human pathogens, such as *E. coli* O157:H7 [27] and *Campylobacter spp*. [28]. 

Other advances include the microfluidics and nanobiotechnological field, allowing the construction of high-density and low-volume qPCR platforms [29]. Droplet Digital PCR (ddPCR) technology uses a combination of microfluidics and proprietary surfactant chemistries to divide PCR samples into water-in-oil droplets [30].

A summary of the application of these techniques for the detection of waterborne pathogens has been presented at the International Scientific Symposium. A study performed by Kabiru *et al.,* [31] evaluated the presence of diarrheagenic *E. coli* in specimens taken at an abattoir located in the Zaria region, Nigeria, in samples of water from the river Koreye and in vegetable specimens taken at a nearby farm. All the isolated *E. coli* were assayed for the production of Shiga toxins (Stx) by using the Ridascreen verotoxin Immunoassay and by PCR amplification of genes associated with the diarrheagenic *E. coli*. The use of these techniques provided evidence of the pathogenic *E. coli* contamination in the environment as a result of the discharge of untreated abattoir effluents.

The study by Jacob *et al*., [32] investigated the occurrence of microorganisms in three rivers utilized both for recreational activities and for drinking water treatment plants through a two-year monitoring campaign. Water samples were concentrated using hollow-fiber ultrafiltration and analyzed by PCR and by laser-scanning cytometry. Health risks related to the presence of *Cryptosprodium parvum*, *Giardia duodenalis*, *Escherichia coli*, *Clostridium perfringens* and adenovirus by accidental ingestion of river water during recreational activities, were assessed through exponential dose-response models. The highest concentrations of protozoan parasites and *C. perfringens* were found for one of the three sites, which may be partly attributable to soil leaching due to rainfall events. The highest concentrations of adenoviruses were detected instead at the two other sites, probably due to strong urban activities.

Finally, Panaiotov *et al*. [33] evaluated the water quality of the Black Sea and the Dam of Iskar by applying molecular tests for the detection of viruses, bacteria and protozoa during the years 2012–2014. The approach used microscopic and PCR analyses for key-pathogens. The results of these authors showed the presence of *Vibrio* spp. in the Black Sea. Rotavirus A was also identified in four samples from the Dam of Iskar. Toxigenic *E. coli* was present in both locations, while no *Mycobacterium* species were present in the water samples. No detectable amounts of *Cryptosporidium* were detected in either locations using immunomagnetic separation and fluorescence microscopy.

DNA sequencing can be used for highly precise and accurate identification of microorganisms at the species level. However, even the use of automated extraction, PCR and DNA sequencing requires time-consuming procedures consisting in the preliminary steps of isolation of the species of interest [4]. The paper by Montalbano *et al.* [34] describes a study to evaluate the presence of free-living amoebae (FLA). Identification of FLA species/genotypes, based on the 18S rDNA regions, allowed to identify 18 (39.1%) *Acanthamoeba* isolates (genotypes T4 and T15) and 21 (45.6%) *Vermamoeba vermiformis* isolates. Other FLA species, including *Vahlkampfia sp*. and *Naegleria spp*., previously reported in Italy, were not detected. The occurrence of potentially pathogenic free-living amoebae in habitats related to human population, supports the relevance of FLA as a potential health threat to humans.

In the study presented by Marcheggiani *et al*. [8], the authors developed and validated a tool for the detection of pathogens in fresh water systems using microarray technology. The results of this study showed the spread of emerging and re-emerging pathogens, such as *Staphylococcus* spp., *Campylobacter* spp., *Clostridium* spp., *Salmonella* spp. and viruses in surface waters close to an urban area. The concentration of cells determined by traditional methods at each sampling site was also compared with the results (signal intensity) of probes spotted on the microarray chip for each microorganism under investigation. Among the main advantages of this methodology are the speed and the possibility of using directly the environmental samples without the need for bacterial cultivation, making them superior to traditional methods. Thus, the microarray technology constitutes a great opportunity for the simultaneous detection of large number of pathogens and may allow the implementation of preventive measures to mitigate their impact on human health.

### 3.2. Bioinformatics Tools

The paper by Singh *et al.* [35] describes the development of a bioinformatics tool for the management of the vast amount of data deriving from microarray experiments involving environmental samples. SaDA, the software described in this paper, is composed of two layers, layer 1 for data upload processing and retrieval and layer 2 for data storage. The advantage of SaDA over existing tools is the flexible and intuitive user interface, which makes this infrastructure easy to handle by users with basic informatics knowledge but with the burden of collecting and storing very large dataset, from sampling to nucleic acid extraction and analyses of microarray hybridization signals. In addition, SaDA is very flexible and extensible; in fact, it easily allows the addition of modules for targeted customization. This aspect is remarkably important in the fast evolving genomic era where high-throughput technologies provide a bevy of data at very high rate.

## 4. Detection of Endocrine Disrupting Chemicals in Water and Liquids

The large numbers of compounds that act as endocrine disrupting compounds (EDCs), their persistence in the environment and the multitude of possible biological effects with disruption of normal hormonal function has led to concerns over their potential for detrimental environmental and health impacts and highlighted a need for their monitoring. Endocrine disruption is thought to play a role in a range of issues in aquatic animals such as intersex and feminisation of fish and may also pose a broader risk to animal and human health including reduced fertility, increased mastitis in livestock, development of abnormalities and increased cancer risks in humans. Consequently, detection of these contaminants in food or water is important not only to protect sensitive environmental sites and reduce the risk of toxins entering the food supply but as a direct measure to protect human health (Radar website http://www.fp7-radar.eu/).

RADAR is a 7-member consortium which, over the past four years, developed a robust, sensitive, and versatile biochemical sensor platform for spot measurements and on-line monitoring of toxins and pollutants, with a focus on EDCs and Polycyclic Aromatic Hydrocarbons (PAHs), in food production processes as well as in the aquatic environment.

One originality of the project was the design and production of aquatic organisms derived receptors *i.e.*, proteins, that recognize and respond to a specific class of pollutants and toxins. These receptors were designed on the basis of the *in vivo* occurring receptors affected by the presence of the class of pollutants targeted. These receptors, namely the estrogen receptor (ER) and the Aryl Hydrocarbon Receptor (AhR), are sensitive not only to a single toxic molecule, but to an entire class of potentially hazardous molecules. A study performed by Pedotti *et al.* [36] used a natural estrogen receptor to monitor the presence of chemical pollutants affecting the endocrine system in water and food samples. EDCs bind to the estrogen receptor and alter its natural functions, causing disease ranging from sexual changes to cancer in animals and humans. The researchers designed a bio-sensor that generates a signal when a ligand binds to laboratory produced estrogen receptor; signal detection indicates the presence of a molecule capable of binding to ER, and thus a likely EDC pollutant. Experimentally validated computational simulations were employed to rationally alter the ER (single point mutations in the EDC binding pocket) in order to increase its affinity for selective classes of EDCs, resulting in increased sensitivity of the biosensor. The RADAR platform, when combined with its wireless communication module, can perform label-free, robust, specific and sensitive detection of toxins and pollutants and to send an alarm signal to a remote control station.

## 5. Conclusions

The Scientific Symposium "*Small solution for big water- related problems: innovative microarrays and small sensores to cope with water quality and food security*" was a conference with more than 100 participants with broad experience on environment and human health issues. Among the several aspects discussed during the Symposium some innovative approaches related to water quality evaluation were presented. These methods included microchip technology; rapid and efficient tools to monitor the presence of emerging and re-emerging pathogens in aquatic ecosystems and the detection of endocrine disrupting chemicals in water. These topics are highly relevant in light of the pressure exerted by the increased impact of human activities as well as by the population growth on the quality and quantity of water resources and their access. It is well recognized that water plays a fundamental role in the transmission of contaminants to human being. Thus, strategic activities focused on environmental resource protection and management (e.g., agricultural and wastewater management practices) may restraint, to some extent, the spread of water-transmitted infectious diseases.

There are still many unsolved issues on safeguard to human health regarding waterborne contaminants. In fact, there are no standard techniques to isolate all pathogens; currently available detection methods are complex, time-consuming and with low reproducibility, the survival of isolated microorganisms is often limited because of the lack of unknown nutrients and the technical complications due to set-up of artificial growth conditions. In addition, the low concentration of pathogens in freshwaters may hamper their detection.

The aim of the two EC-funded Projects moves toward the development and the implementation of sensitive techniques for the detection of different types of pathogens and their toxins. The results presented at the Symposium showed how the new tools (microarray chips and small sensors) are capable of providing innovative approaches for monitoring the environment and for establishing the correlation between the health of an ecosystem and human health.

## Appendix

Useful/related links:
http://www.microaqua.eu
http://www.fp7-radar.eu
http://innovativeaquaticbiosensors.com/
http://www.iss.it/itti/index.php?lang=1&id=148&tipo=15
http://www.iss.it/binary/publ/cont/14_C6.pdf
http://www.euronews.com/2014/06/30/is-it-safe-seeking-a-universal-test-for-fresh-water/

(The movie has been translated in thirteen different languages to be reached from more than 200 millions of people in the world).
